# Fetal growth restriction as the initial finding of preeclampsia is a clinical predictor of maternal and neonatal prognoses: a single-center retrospective study

**DOI:** 10.1186/s12884-021-04152-2

**Published:** 2021-10-06

**Authors:** Masaya Takahashi, Shintaro Makino, Kyoko Oguma, Haruka Imai, Ai Takamizu, Akari Koizumi, Koyo Yoshida

**Affiliations:** Department of Obstetrics and Gynecology, Juntendo University Urayasu Hospital, Tomioka 2-1-1, Urayasu City, Chiba, 279-0021 Japan

**Keywords:** Preeclampsia, Hypertensive disorder of pregnancy, Fetal growth restriction, New ISSHP criteria, Eclampsia, Cesarean section, Nonreassuring fetal status, Pregnancy complications, Adverse pregnancy outcomes

## Abstract

**Background:**

Preeclampsia (PE) is a hypertensive disorder specific to pregnancy that can cause severe maternal-neonatal complications. The International Society for the Study of Hypertension in Pregnancy revised the PE criteria in 2018; a PE diagnosis can be established in the absence of proteinuria when organ or uteroplacental dysfunction occurs. The initial findings of PE (IFsPE) at the first diagnosis can vary considerably across patients. However, the impacts of different IFsPE on patient prognoses have not been reported. Thus, we investigate the predictors of pregnancy complications and adverse pregnancy outcomes based on IFsPE according to the new criteria.

**Methods:**

This retrospective study included 3729 women who delivered at our hospital between 2015 and 2019. All women were reclassified based on the new PE criteria and divided into three groups based on the IFsPE: *Classification 1* (*C-1*), proteinuria (classical criteria); *Classification 2* (*C-2*), damage to other maternal organs; and *Classification 3* (*C-3*), uteroplacental dysfunction. Pregnancy complications and adverse pregnancy outcomes were assessed and compared among the three groups.

**Results:**

In total, 104 women with PE were included. Of those, 42 (40.4%), 28 (26.9%), and 34 (32.7%) were assigned to *C-1*, *C-2*, and *C-3* groups, respectively. No significant differences in maternal characteristics were detected among the three groups, except for gestational age at PE diagnosis (*C-1*, 35.5 ± 3.0 weeks; *C-2*, 35.2 ± 3.6 weeks; *C-3*, 31.6 ± 4.6 weeks, *p* <  0.01). The rates of premature birth at < 37 weeks of gestation, fetal growth restriction (FGR), and neonatal acidosis were significantly higher in the *C-3* group compared to the *C-1* and *C-2* groups. Additionally, the composite adverse pregnancy outcomes of the *C-3* group compared with *C-1* and *C-2* represented a significantly higher number of patients.

**Conclusions:**

PE patients with uteroplacental dysfunction as IFsPE had the most unfavorable prognoses for premature birth, FGR, acidosis, and composite adverse pregnancy outcomes.

**Supplementary Information:**

The online version contains supplementary material available at 10.1186/s12884-021-04152-2.

## Background

Preeclampsia (PE) is a hypertensive disorder that occurs during pregnancy, with a reported incidence of 3–8% [1]. As a major cause of premature birth, PE can threaten both maternal and fetal/neonatal life, accounting for more than 50,000 maternal and 500,000 neonatal deaths annually worldwide [2]. The detailed and exact etiology of PE remains unknown. However, inadequate trophoblast invasion and angiogenesis, resultant inappropriate remodeling of uterine spiral arteries, and increased production of antiangiogenic factors, such as soluble fms-like tyrosine kinase 1 (sFlt-1) and soluble endoglin (sEng), have been identified as crucial contributors [3, 4]. These inappropriate responses can lead to uteroplacental dysfunction and subsequent maternal endothelial dysfunction, contributing to the development of PE. Uteroplacental dysfunction, which causes placental hypoxic conditions and nutritional deficiency, leads to fetal growth restriction (FGR). Although various studies have investigated treatments for suppressing PE progression [5], a practical and effective treatment has yet to be identified. Thus, delivery is currently the only treatment option for PE.

In 2018, the International Society for the Study of Hypertension in Pregnancy (ISSHP) revised the definitions of PE and categorized PE into three classes: PE with proteinuria (classical criteria), dysfunction of other maternal organs, and uteroplacental dysfunction [6]. Thus, the initial findings of PE (IFsPE) can vary considerably across patients at the time of diagnosis. Therefore, we hypothesized that evaluating and differentiating clinical features according to each IFsPE through risk classification may enable appropriate IFsPE-based management. However, the impacts and adverse clinical outcomes associated with different IFsPE have not been reported. Thus, our objective is to identify the predictors of pregnancy complications and adverse pregnancy outcomes based on IFsPE according to the new ISSHP criteria.

## Methods

### Study design and subjects

**Fig. 1:**
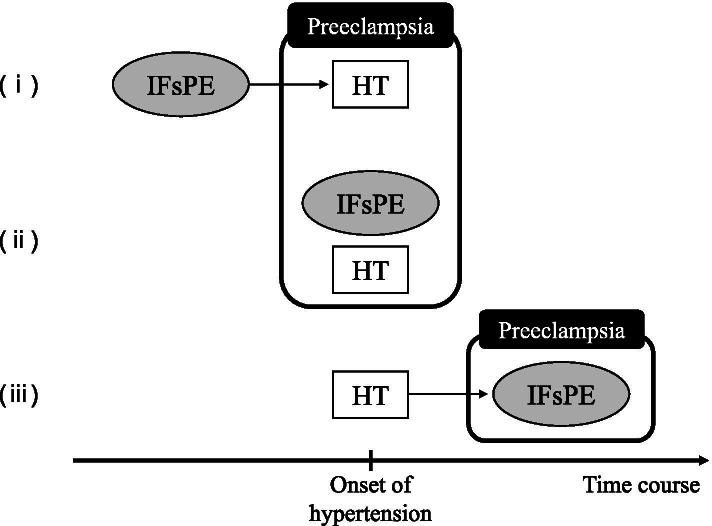
Schematic images of time course at the time of preeclampsia diagnosis. All patients were diagnosed with PE based on the following three conditions: (i) IFsPE complicated later by hypertension, (ii) simultaneous onset of both IFsPE and hypertension, and (iii) hypertension complicated later by IFsPE. PE, preeclampsia; IFsPE, initial findings of the PE; HT, hypertension

A retrospective cohort study was designed with approval from the Ethical Committee of Juntendo University Urayasu Hospital (No. 2020–044). The medical records of mothers and neonates were obtained from our electronic medical database. This study included pregnant women who delivered at our teaching hospital between January 2015 and December 2019. Patients with PE were reclassified based on the new ISSHP criteria and divided into three groups based on IFsPE according to the ISSHP classifications: *Classification 1* (*C-1*), proteinuria (classical criteria); *Classification 2* (*C-2*), damage to other maternal organs; and *Classification 3* (*C-3*), uteroplacental dysfunction. As shown in Fig. 1, all patients were diagnosed with PE under one of three circumstances: (i) IFsPE complicated later by hypertension, (ii) simultaneous onset of IFsPE and hypertension, or (iii) hypertension complicated later by IFsPE.

### Diagnosis and management of preeclampsia

Patients were classified using the new 2018 ISSHP criteria to diagnose PE [6]. Hypertension was defined as systolic blood pressure > 140 mmHg or diastolic blood pressure > 90 mmHg when measured on at least two occasions. According to the new criteria, PE can be diagnosed in the absence of proteinuria (defined as a protein level > 300 mg/day over 24 h or a measured urinary protein to creatinine ratio > 0.3) when maternal organ dysfunction or uteroplacental dysfunction occur. Maternal organ dysfunction includes renal insufficiency, liver involvement with or without right upper quadrant involvement or epigastric abdominal pain, neurological complications (altered mental status, blindness, stroke, clonus, severe headaches, persistent visual scotomata), and hematological complications (platelet counts < 150,000/μL, disseminated intravascular coagulation, hemolysis). Uteroplacental dysfunction includes FGR, abnormal umbilical artery (UA) Doppler waveforms, and stillbirth. In ultrasound examinations, the fetal biparietal diameter (BPD) and femur length (FL) were measured using the linear function, and abdominal circumference (AC) was measured using the ellipse function according to the standard techniques outlined by the Japan Society of Ultrasonics in Medicine (JSUM) [7]. The ultrasonography equipment automatically estimated the fetal weight (EFW) using the formula proposed by the JSUM: EFW (g) = 1.07 × BPD (cm)^3^ + 0.30 × AC (cm)^2^ × FL (cm). FGR was diagnosed if the EFW was below the 10th percentile for gestational age according to the Japanese fetal growth curve [7]. Ultrasound examinations and nonstress tests were performed at least once a week. Blood pressure was evaluated at least three times daily, and blood samples were collected at least once a week. Rest was prescribed to the patients, and oral antihypertensive drugs, such as methyldopa, calcium blockers, α- and β-blockers, and intravenous calcium blockers, were administered if necessary. Magnesium sulfate (MgSO_4_) was administered to patients with eclampsia or high risk of eclampsia to prevent seizures. Initially, 4 g of MgSO_4_ was administered intravenously for 30 min, followed by a continued dose of 1 g/h and close monitoring of the serum magnesium level and related side effects. Antenatal betamethasone was administered to mothers to promote fetal lung maturation before 34 weeks of gestation. Delivery was indicated by the inability to control maternal blood pressure using antihypertensive drugs; placental abruption; eclampsia; hemolysis, elevated liver enzymes, and low platelet count (HELLP) syndrome; nonreassuring fetal status (NRFS); fetal growth arrest lasting 2 weeks; stillbirth; or 37 weeks of gestation. The attending physician selected the mode of delivery according to obstetric and fetal conditions.

To investigate differences in clinical features and maternal/neonatal prognoses among the three groups based on IFsPE (*C-1*, *C-2*, and *C-3*), the following items were evaluated and compared: maternal age, pre-pregnancy body mass index (BMI), pregnancy history, the indication of delivery (maternal or fetal factors), and gestational age at PE diagnosis. Pregnancy complications, including premature birth at < 37 weeks, FGR, NRFS, and placental abruption, were assessed. Adverse pregnancy outcomes, including acidosis (defined as UA pH < 7.20), Apgar score at 5 min < 7, stillbirth, neonatal mortality, and composite adverse pregnancy outcomes, were recorded. Composite adverse pregnancy outcomes were defined as the presence of one or more of the following conditions: acidosis, Apgar score at 5 min < 7, stillbirth, and neonatal mortality.

### Statistical analysis

Statistical analyses were performed using SPSS 18.0 for Windows (SPSS Inc., Chicago, IL). Differences between two groups were evaluated using Student’s *t*-test for continuous variables and chi-square or Fisher’s exact tests for categorical variables. Differences among three groups were analyzed using one-way analysis of variance with multiple comparisons, followed by a *post-hoc* Tukey’s test for continuous variables and Fisher’s exact test for categorical variables. The complication risks in each group are presented as crude odds ratios (ORs) with 95% confidence intervals (95% CIs). *P*-values < 0.05 were considered statistically significant. All data are presented as the mean ± SD or *n* (%).

## Results

**Fig. 2:**
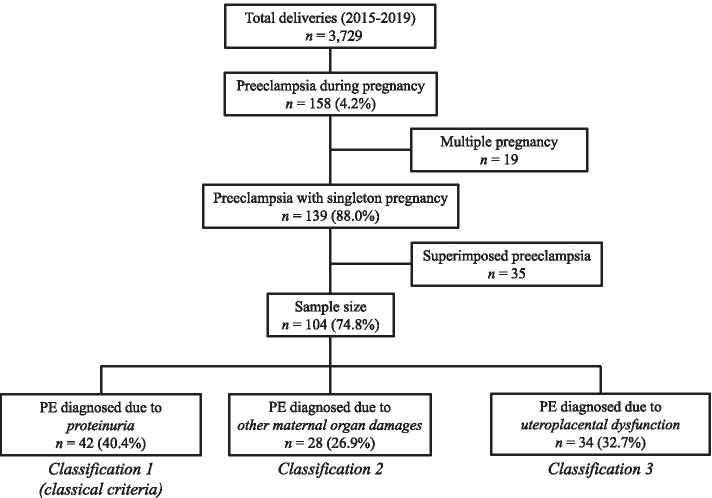
Flow diagram of the inclusion of study participants from 2015 to 2019. All women were reclassified based on the ISSHP criteria. They were categorized based on the initial findings of preeclampsia (PE) at the first diagnosis into three groups according to the ISSHP categories: *Classification 1 (classical criteria)*, PE diagnosed based on proteinuria; *Classification 2*, PE diagnosed based on damage to maternal organs; and *Classification 3*, PE diagnosed based on uteroplacental dysfunction. ISSHP, International Society for the Study of Hypertension in Pregnancy; PE, preeclampsia.

Among 3729 women who delivered at our teaching hospital between January 2015 and December 2019, 158 (4.2%) were diagnosed with PE. After excluding 19 cases of multiple pregnancies, 139 (88.0%) single pregnancy PE cases remained. An additional 35 cases were excluded on the basis of superimposed PE. The remaining 104 (74.8%) cases were included in our analysis (Fig. 2).

### Comparison of pregnancy complications and adverse pregnancy outcomes among C-1, C-2, and C-3

The baseline characteristics of the three groups (*C-1* vs. *C-2* vs. *C-3*) are presented and compared in Table 1. Of 104 subjects, 42 (40.4%), 28 (26.9%), and 34 (32.7%) were classified into the *C-1*, *C-2*, and *C-3* groups, respectively. No significant differences in maternal age, pre-pregnancy BMI (including a BMI > 25 kg/m^2^), primiparous rate, and indication of delivery were detected among the three groups. However, the gestational age at PE diagnosis was significantly lower in the C-3 group.

**Table 1:** Comparison of maternal characteristics across the three groups based on ISSHP categories

	*Classification 1* (*n* = 42)			*Classification 2* (*n* = 28)			*Classification 3* (*n* = 34)			*P* value
Maternal age (years)	33.8	±	4.9	33.9	±	5.4	35.0	±	6.2	n.s.
Pre-pregnancy BMI	23.4	±	4.4	23.0	±	3.6	22.1	±	3.1	n.s.
Obesity (BMI > 25 kg/m^2^)	13 (31.0%)			7 (25.0%)			6 (17.6%)			n.s.
Primiparity	32 (76.2%)			16 (57.1%)			23 (67.6%)			n.s.
Indication of delivery										
Maternal factors	33 (78.6%)			21 (75.0%)			22 (64.7%)			n.s.
Fetal factors	9 (21.4%)			7 (25.0%)			12 (35.3%)			
Gestational age at the PE diagnosis (weeks)	35.5	±	3.0	35.2	±	3.6	31.6	±	4.6	< 0.0001

Abbreviations: BMI, body mass index; PE, preeclampsia; n.s., not significant; SD, standard deviation; ISSHP, International Society for the Study of Hypertension in Pregnancy

Data are presented as mean ± SD or n (%)

P-values < 0.05 were considered as statistically significant

**Fig. 3:**
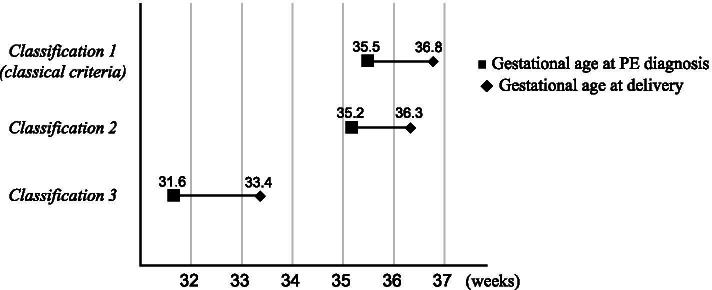
Timing of PE onset and the timing of delivery among the three groups PE, preeclampsia

Pregnancy complications across the three groups are presented and compared in Table 2. The rate of premature birth at < 37 weeks of gestation was significantly higher in *C-3* than in *C-1* and *C-2*. The temporal relationship between the time of PE diagnosis and delivery is illustrated in Fig. 3. The percentage of patients with FGR was also significantly higher (*p* <  0.0001) in the *C-3* group [34 (100.0%)] than in *C-1* [7 (16.7%)] and *C-2* [4 (14.3%)] groups. No significant differences in the rates of NRFS and placental abruption were detected among groups.

**Table 2:** Comparison of pregnancy complications across the three groups based on ISSHP categories

	*C-1*	*C-2*	*C-3*	*C-1* vs. *C-3*	*C-2* vs. *C-3*	*C-1* vs. *C-3*		*C-2* vs. *C-3*	
	(n = 42)	(n = 28)	(n = 34)	P value	P value	OR	95% CI	OR	95% CI
Premature birth at < 37 weeks	20 (47.6%)	13 (46.4%)	26 (76.5%)	< 0.05	< 0.05	3.58	1.32–9.69	3.75	1.27–11.11
FGR	7 (16.7%)	4 (14.3%)	34 (100.0%)	< 0.0001	< 0.0001	–	–	–	–
NRFS	16 (38.1%)	13 (46.4%)	20 (58.8%)	n.s.	n.s.	2.32	0.92–5.85	1.65	0.60–4.52
Placental abruption	1 (2.4%)	1 (3.6%)	3 (8.8%)	n.s.	n.s.	3.97	0.39–40.00	2.61	0.26–26.62

Abbreviations: C-1, classification 1; C-2, classification 2; C-3, classification 3; FGR, fetal growth restriction; NRFS, nonreassuring fetal status; OR, odds ratio; CI, confidence interval; n.s., not significant; ISSHP, International Society for the Study of Hypertension in Pregnancy

FGR was diagnosed if the estimated fetal weight determined by ultrasonography was <10th percentile for gestational age according to the Japanese fetal growth curve

Data are presented as n (%), or OR with 95% CI

P-values < 0.05 were considered statistically significant

Adverse pregnancy outcomes across the three groups are presented and compared in Table 3. The number of patients with neonatal acidosis was significantly higher in *C-3* than in *C-1* and *C-2* groups. Apgar scores < 7 at 5 min, stillbirth, and neonatal mortality could not be compared among groups due to the small sample sizes; thus, we assessed the composite of these adverse pregnancy outcomes. Significantly more patients in the *C-3* group experienced composite adverse pregnancy outcomes compared to those in the other groups.

**Table 3:** Comparison of adverse pregnancy outcomes across the three groups based on ISSHP categories

	*C-1*	*C-2*	*C-3*	*C-1* vs. *C-3*	*C-2* vs. *C-3*	*C-1* vs. *C-3*		*C-2* vs. *C-3*	
	(n = 42)	(n = 28)	(n = 34)	P value	P value	OR	95% CI	OR	95% CI
Acidosis	3(7.1%)	2(7.1%)	9 (26.5%)	< 0.05	< 0.05	4.68	1.15–18.98	4.68	0.92–23.83
Apgar score (5 min) < 7	2 (4.8%)	0 (0%)	6 (17.6%)	n.s.	–	4.29	0.81–22.80	–	–
Stillbirth	0	0	1 (2.9%)	–	–	–	–	–	–
Neonatal mortality	0	0	1 (2.9%)	–	–	–	–	–	–
Composite adverse pregnancy outcomes	5 (11.9%)	2 (7.1%)	12 (35.5%)	< 0.05	< 0.005	4.04	1.25–12.99	7.09	1.43–35.16

Abbreviations: C-1, classification 1; C-2, classification 2; C-3, classification 3; OR, odds ratio; CI, confidence interval; n.s., not significant; ISSHP, International Society for the Study of Hypertension in Pregnancy

Acidosis is defined as umbilical artery pH < 7.20. The composite adverse pregnancy outcomes include acidosis, Apgar score < 7 at 5 min, stillbirth, and neonatal mortality

Data are presented as n (%), or OR with 95% CI

P-values < 0.05 were considered statistically significant

## Discussion

Our findings demonstrated that patients diagnosed with PE in whom the IFsPE was uteroplacental dysfunction (*C-3* group) had significantly lower gestational ages at delivery and higher rates of FGR, acidosis, and composite adverse pregnancy outcomes than those in the other two groups. Thus, patients classified into the *C-3* group showed the most unfavorable prognoses in terms of adverse pregnancy outcomes, indicating that special attention and more careful management are necessary for *C-3* PE patients.

We identified the clinical prognostic factors among the three classification groups of patients with PE, which provides useful information for managing PE and explaining the medical condition and associated risks to patients and their families. To the best of our knowledge, this is the first study to compare pregnancy complications and adverse pregnancy outcomes among the three new ISSHP categories based on IFsPE. However, due to the small sample size, we were unable to classify the IFsPE of maternal organ dysfunction (such as renal insufficiency, liver involvement, neurological complications, and hematological complications) in the *C-2* group in detail. Additional case data is needed to compare detailed outcomes and clarify the differences in prognoses across groups to establish guidelines for IFsPE-based management of PE in the future.

In 2018, the ISSHP revised its definitions of PE [6]. In contrast to the ISSHP criteria, the 2013 American College of Obstetricians and Gynecologists PE diagnostic criteria do not include uteroplacental dysfunction because it should be managed similarly in patients with or without PE [8]. FGR is the most common uteroplacental dysfunction, representing all patients in the C-3 group in our cohort. FGR is associated with an increased risk of maternal and neonatal complications in PE patients [9–11]. A previous study revealed that the gestational age at delivery was significantly lower, and the rates of maternal complications and neonatal adverse outcomes were significantly higher in PE patients with FGR than those without FGR [9, 10]. In contrast, another study concluded that gestational age at delivery and maternal complication rates were similar among PE patients with and without FGR; however, a higher risk of intrauterine fetal death (a neonatal adverse outcome) was associated with FGR [11]. Based on these findings, it is unclear whether PE patients with FGR are more likely to experience maternal complications than PE patients without FGR. As uteroplacental dysfunction is a significant etiology of PE, it is an acceptable diagnostic criterion for PE. In addition, Mitani et al. [10] reported that approximately 15% of patients with FGR experienced proteinuria as a complication, which was diagnosed as PE, suggesting that patients with FGR required close monitoring for PE detection.

While the exact etiology of PE remains unclear, a two-stage disorder theory for the etiology and pathology of PE was recently proposed [12, 13]. Evidence-based methods of prevention or treatment have not yet been established; therefore, it is clinically important to be aware of early PE-related symptoms or findings, which can widely vary among patients, allowing for appropriate and early management based on risk classification. Our investigation is the first to focus on adverse pregnancy outcomes of PE patients classified according to the new ISSHP criteria based on IFsPE. We compared adverse pregnancy outcomes among *C-1*, *C-2*, and *C-3* groups. Poor prognoses were observed in patients in the *C-3* group, including higher risks of lower gestational age at delivery, FGR, and composite adverse pregnancy outcomes in the *C-3* group than in the other two groups. In addition, the number of patients with neonatal acidosis was significantly higher in the *C-3* group compared to the other groups. These findings indicate that special attention, such as additional gynecological checkup, should be paid when patients are diagnosed with PE based on uteroplacental dysfunction as the IFsPE in the following situations: uteroplacental dysfunction complicated later by hypertension, simultaneous onset of uteroplacental dysfunction and hypertension, and hypertension complicated later by uteroplacental dysfunction.

As shown in Table 1, most PE patients with FGR were diagnosed with PE based on FGR as the IFsPE. However, antiangiogenic factor production might increase with worsening uteroplacental circulation, subsequently inducing FGR and exacerbating maternal endothelial dysfunction. Previous studies have demonstrated that sFlt-1 levels, sEng levels, and the sFlt-1 to placental growth factor (PIGF) ratio (sFlt-1/PIGF) were significantly increased in PE patients with FGR compared to those in patients without FGR [14, 15], which supports our conjecture. Further research is required to clarify the relationship between uteroplacental dysfunction and maternal endothelial dysfunction.

Moreover, the UA O_2_ level was significantly lower in PE patients with FGR than in those without FGR and in PE patients categorized into the *C-3* group than those in the *C-1* and *C-2* groups (Supplementary Table S1). Therefore, we speculated that patients with FGR had underlying chronic fetal hypoxia due to uteroplacental dysfunction, which limited the gas and nutrient exchange and caused the resultant FGR. In recent years, the application of hemoglobin vesicles to treat conditions such as brain ischemia and massive obstetric hemorrhage has been suggested based on the results of animal model experiments [16–18]. Heng Li et al. [19] demonstrated that artificial nano-oxygen carriers can be used to successfully treat placental hypoxia and manage FGR and apoptotic damage in the brain using a PE rat model. Given our results, this noninvasive therapy could potentially delay the progression of PE and improve neonatal outcomes. However, if lower UA O_2_ levels are caused by fetal conditions such as NRFS rather than underlying chronic fetal hypoxia, this treatment option would not be viable.

The limitations of this study should be acknowledged. Our results may reflect the differences between gestational age at diagnosis. We understand the necessity of adjustment for gestational age; however, our small sample size did not allow us to perform a multiple logistic regression. Our results, especially the significantly higher rates of pregnancy complications, such as premature birth and FGR, in the *C-3* group, may be related to the earlier onset of PE. Regardless, our findings suggest that pregnant women with uteroplacental dysfunction as the IFsPE may require extra attention during pregnancy due to a higher risk for earlier onset of PE. Additionally, this was a single-center retrospective cohort study with a small sample size, which might have affected the results of the study. In particular, the sample sizes of various *C-2* group IFsPE types, such as renal insufficiency, liver involvement, neurological complications, and hematological complications, were small; therefore, we could not clarify the differences based on detailed IFsPE. To establish detailed guidelines for the IFsPE-based management of PE, a study with a large sample size is required to verify the accuracy of our results and detect differences in prognoses across the detailed IFsPE groups. Finally, our hospital is a perinatal medical center, and severe PE patients were likely to be transferred, which may result in some differences compared with general hospitals.

This is the first study to compare maternal and neonatal prognoses among the three new ISSHP categories based on IFsPE. All data were electronically recorded, and patients were retrospectively reclassified and diagnosed with PE according to the 2018 ISSHP criteria, preventing selection bias. We showed that PE patients presenting with uteroplacental dysfunction as the IFsPE had poor prognoses, especially for outcomes related to perinatal health, such as premature birth, FGR, acidosis, and composite adverse pregnancy outcomes. Our findings suggest that IFsPE may be a predictor of perinatal prognosis.

## Conclusions

In summary, our study suggested that more unfavorable prognoses, including higher risks of premature birth, FGR, acidosis, and composite adverse pregnancy outcomes, were observed in PE patients classified in the *C-3* group (presenting with uteroplacental dysfunction as the IFsPE) than in other groups. Thus, special attention and more careful management are necessary for PE patients categorized into the *C-3* group. Moreover, careful and close observation, especially in terms of the adverse outcomes related to perinatal health, is required in patients presenting with uteroplacental dysfunction prior to PE diagnosis. Our results are useful in terms of explaining the expected prognosis of PE to patients and their families. Additional studies with larger sample sizes should be conducted to confirm our findings.

## Supplementary Information

**Additional file 1.**

## Data Availability

The datasets used and/or analyzed during the study are available from the corresponding author upon reasonable request.

## Abbreviations

AC: Abdominal circumference
BMI: Body mass index
BPD: Biparietal diameter
EFW: Estimated the fetal weight
FGR: Fetal growth restriction
FL: Femur length
HELLP: Hemolysis, elevated liver enzymes, and low platelet
IFsPE: initial findings of preeclampsia
ISSHP: International Society for the Study of Hypertension in Pregnancy
NRFS: Nonreassuring fetal status
OR: Odds ratios
PE: Preeclampsia
PIGF: Placental growth factor
UA: Umbilical artery

## References

[1] Carty DM, Delles C, Dominiczak AF (2010). Preeclampsia and future maternal health. J Hypertens.

[2] Whorld Health Organization. The world health report: 2005: make every mother and child count. GenevaWHO2005. Available at: http://www.who.int/whr/2005/whr2005_en.pdf. Accessed November 1, 2020.

[3] Aneman I, Pienaar D, Suvakov S, Simic TP, Garovic VD, McClements L (2020). Mechanisms of key innate immune cells in early- and late-onset preeclampsia. Front Immunol.

[4] Seki H (2014). Balance of antiangiogenic and angiogenic factors in the context of the etiology of preeclampsia. Acta Obstet Gynecol Scand.

[5] Grimes S, Bombay K, Lanes A, Walker M, Corsi DJ (2019). Potential biological therapies for severe preeclampsia: a systematic review and meta-analysis. BMC Pregnancy Childbirth.

[6] Brown MA, Magee LA, Kenny LC, Karumanchi SA, McCarthy FP, Saito S (2018). Hypertensive disorders of pregnancy: ISSHP classification, diagnosis, and management recommendations for international practice. Hypertension..

[7] Okai T (2003). Standardization for ultrasonographic measurement of fetus and Japanese standard. Jpn J Med Ultrasonics.

[8] American College of, O., Gynecologists, and P. Task Force on Hypertension in, Hypertension in pregnancy. Report of the American College of Obstetricians and Gynecologists' Task Force on Hypertension in Pregnancy. Obstet Gynecol. 2013;122(5):1122–31. 10.1097/01.AOG.0000437382.03963.88 24150027

[9] Balogun OA, Khanagura RK, Kregel HR, Amro FH, Sibai BM, Chauhan SP (2018). Preterm preeclampsia with severe features: composite maternal and neonatal morbidities associated with fetal growth restriction. Am J Perinatol.

[10] Mitani M, Matsuda Y, Makino Y, Akizawa Y, Ohta H (2009). Clinical features of fetal growth restriction complicated later by preeclampsia. J Obstet Gynaecol Res.

[11] Haddad B, Kayem G, Deis S, Sibai BM. Are perinatal and maternal outcomes different during expectant management of severe preeclampsia in the presence of intrauterine growth restriction? Am J Obstet Gynecol. 2007;196(3):237 e1–5. 10.1016/j.ajog.2006.10.905 17346535

[12] Roberts JM (2000). Preeclampsia: what we know and what we do not know. Semin Perinatol.

[13] Roberts, J.M. and C.A. Hubel. The two stage model of preeclampsia: variations on the theme. Placenta. 2009;30 Suppl A:S32–7. 10.1016/j.placenta.2008.11.009 PMC2680383 19070896

[14] Nanjo S, Minami S, Mizoguchi M, Yamamoto M, Yahata T, Toujima S (2017). Levels of serum-circulating angiogenic factors within 1 week prior to delivery are closely related to conditions of pregnant women with pre-eclampsia, gestational hypertension, and/or fetal growth restriction. J Obstet Gynaecol Res.

[15] Zhao W, Qiao J, Zhang Q, Zhao Y, Chen Q (2010). Levels of antiangiogenic factors in preeclamptic pregnancies. Growth Factors.

[16] Chang TM (2005). Therapeutic applications of polymeric artificial cells. Nat Rev Drug Discov.

[17] Fukumoto D, Kawaguchi AT, Haida M, Yamano M, Ogata Y, Tsukada H (2009). Liposome-encapsulated hemoglobin reduces the size of cerebral infarction in rats: effect of oxygen affinity. Artif Organs.

[18] Yuki Y, Hagisawa K, Kinoshita M, Ishibashi H, Kaneko K, Ishida O, et al. Efficacy of resuscitative infusion with hemoglobin vesicles in rabbits with massive obstetric hemorrhage. Am J Obstet Gynecol 2020. 10.1016/j.ajog.2020.09.010 32926859

[19] Li H, Ohta H, Tahara Y, Nakamura S, Taguchi K, Nakagawa M (2015). Artificial oxygen carriers rescue placental hypoxia and improve fetal development in the rat pre-eclampsia model. Sci Rep.

