# Supplementary material for: Fetal growth restriction as the initial finding of preeclampsia is a clinical predictor of maternal and neonatal prognoses: a single-center retrospective study
Source: BMC Pregnancy Childbirth. 2021 Oct 6;21:678. doi: 10.1186/s12884-021-04152-2 (PMC8495959; doi:10.1186/s12884-021-04152-2)
Supplement: Supplementary file 1 — Additional file 1. [file 12884_2021_4152_MOESM1_ESM.docx]

Supplementary Table S1. Comparison of maternal and neonatal outcomes

|  | *C-1* | | | *C-2* | | | *C-3* | | |  | *C-1* vs. *C-3* |  | *C-2* vs. *C-3* |  | *C-1* vs. *C-3* | |  | *C-2* vs. *C-3* | |
| --- | --- | --- | --- | --- | --- | --- | --- | --- | --- | --- | --- | --- | --- | --- | --- | --- | --- | --- | --- |
|  | (n = 42) | | | (n = 28) | | | (n = 34) | | |  | P value |  | P value |  | OR | 95% CI |  | OR | 95% CI |
| **Maternal** |  |  |  |  |  |  |  |  |  |  |  |  |  |  |  |  |  |  |  |
| Gestational age at delivery (weeks) | 36.8 | ± | 2.8 | 36.3 | ± | 3.2 | 33.4 | ± | 4.4 |  | < 0.001 |  | < 0.01 |  | - | - |  | - | - |
| Premature birth at < 34 weeks | 6 (14.3%) | | | 7 (25.0%) | | | 17 (50.0%) | | |  | < 0.0001 |  | < 0.0001 |  | 6.0 | 2.01-17.93 |  | 3.00 | 1.01-8.90 |
| Duration from diagnosis to delivery (weeks) | 1.3 | ± | 1.2 | 1.1 | ± | 2.0 | 1.7 | ± | 1.6 |  | n.s. |  | n.s. |  | - | - |  | - | - |
| Mode of delivery |  |  |  |  |  |  |  |  |  |  |  |  |  |  |  |  |  |  |  |
| Cesarean section | 24 (57.1%) | | | 15 (53.6%) | | | 29 (85.3%) | | |  | < 0.05 |  | < 0.05 |  | 4.35 | 1.41-13.45 |  | 5.03 | 1.51-16.78 |
| Instrumental vaginal delivery | 7 (16.7%) | | | 6 (21.4%) | | | 2 (5.9%) | | |  | n.s. |  | n.s. |  | 0.31 | 0.06-1.62 |  | 0.23 | 0.04-1.24 |
| Blood loss (g), median (range) | 650 (165-1820) | | | 555 (111-2060) | | | 498 (99-1300) | | |  | n.s. |  | n.s. |  | - | - |  | - | - |
| Duration of hospital stay | 12.6 | ± | 6.2 | 11.6 | ± | 5.1 | 12.4 | ± | 5.4 |  | n.s. |  | n.s. |  | - | - |  | - | - |
| Eclampsia | 0 | | | 2 (7.1%) | | | 0 | | |  | - |  | - |  | - | - |  | - | - |
| HELLP syndrome | 2 (4.8%) | | | 6 (21.4%) | | | 1 (2.9%) | | |  | n.s. |  | n.s. |  | 0.61 | 0.05-6.98 |  | 0.11 | 0.01-0.99 |
| Blood transfusion | 5 (11.9%) | | | 4 (14.3%) | | | 4 (11.8%) | | |  | n.s. |  | n.s. |  | 0.99 | 0.24-4.00 |  | 0.80 | 0.18-3.54 |
| Pleural effusion or ascites | 15 (35.7%) | | | 4 (14.3%) | | | 9 (26.5%) | | |  | n.s. |  | n.s. |  | 0.65 | 0.24-1.74 |  | 2.16 | 0.59-7.96 |
| **Neonatal** |  | | |  | | |  | | |  |  |  |  |  |  |  |  |  |  |
| Birth weight (g) | 2366 | ± | 617 | 2255 | ± | 666 | 1361 | ± | 646 |  | < 0.0001 |  | < 0.0001 |  | - | - |  | - | - |
| Apgar score (1 min), Median (range) | 8 (2-9) | | | 8 (5-9) | | | 7 (1-9) | | |  |  |  |  |  |  |  |  |  |  |
| < 7 | 5 (11.9%) | | | 5 (17.9%) | | | 16 (47.1%) | | |  | < 0.001 |  | < 0.05 |  | 6.58 | 2.08-20.80 |  | 4.09 | 1.26-13.29 |
| < 4 | 3 (7.1%) | | | 0 (0%) | | | 10 (29.4%) | | |  | < 0.05 |  | - |  | 5.42 | 1.35-21.68 |  | - | - |
| Apgar score (5 min), Median (range) | 9 (5-10) | | | 9 (7-10) | | | 8.5 (4-10) | | |  |  |  |  |  |  |  |  |  |  |
| < 4 | 0 (0%) | | | 0 (0%) | | | 0 (0%) | | |  | - |  | - |  | - | - |  | - | - |
| Umbilical blood analysis |  |  |  |  |  |  |  |  |  |  |  |  |  |  |  |  |  |  |  |
| pH | 7.28 | ± | 0.10 | 7.30 | ± | 0.06 | 7.26 | ± | 0.09 |  | n.s. |  | n.s. |  | - | - |  | - | - |
| O_2_ | 18.8 | ± | 5.6 | 20.9 | ± | 5.0 | 16.9 | ± | 4.8 |  | n.s. |  | < 0.01 |  | - | - |  | - | - |
| CO_2_ | 51.6 | ± | 16.2 | 50.1 | ± | 11.7 | 55.1 | ± | 10.3 |  | n.s. |  | n.s. |  | - | - |  | - | - |
| HCO_3_ | 23.1 | ± | 2.2 | 22.8 | ± | 3.3 | 23.8 | ± | 2.6 |  | n.s. |  | n.s. |  | - | - |  | - | - |
| BE | -4.8 | ± | 5.9 | -4.3 | ± | 4.1 | -4.0 | ± | 3.4 |  | n.s. |  | n.s. |  | - | - |  | - | - |
| Lactate | 33.0 | ± | 14.1 | 35.7 | ± | 15.8 | 37.4 | ± | 20.8 |  | n.s. |  | n.s. |  | - | - |  | - | - |
| Admission to the NICU | 16 (38.1%) | | | 14 (50.0%) | | | 30 (88.2%) | | |  | < 0.0001 |  | < 0.01 |  | 12.19 | 3.62-41.08 |  | 7.50 | 2.09-26.96 |
| Infant mortality | 0 | | | 0 | | | 1 (2.9%) | | |  | - |  | - |  | - | - |  | - | - |
| RDS | 4 (9.5%) | | | 1 (3.6%) | | | 8 (23.5%) | | |  | n.s. |  | n.s. |  | 2.92 | 0.80-10.72 |  | 8.31 | 0.97-71.14 |
| PVL | 0 | | | 0 | | | 1 (2.9%) | | |  | - |  | - |  | - | - |  | - | - |
| ROP | 2 (4.8%) | | | 0 | | | 9 (26.5%) | | |  | < 0.01 |  | - |  | 7.20 | 1.44-36.08 |  | - | - |
| Tracheal intubation | 5 (11.9%) | | | 4 (14.3%) | | | 13 (38.2%) | | |  | < 0.05 |  | < 0.05 |  | 4.58 | 1.43-14.64 |  | 3.71 | 1.05-13.15 |

Abbreviations: C-1, classification 1; C-2, classification 2; C-3, classification 3; HELLP, hemolysis, elevated liver enzymes, and low platelets; NICU, neonatal intensive care unit; RDS, respiratory distress syndrome; PVL, periventricular leukomalacia; ROP, retinopathy of prematurity; OR, odds ratio; CI, confidence interval; n.s., not significant; ISSHP, International Society for the Study of Hypertension in Pregnancy; SD, standard deviation

Data are presented as the mean ± SD, median with range, n (%), or OR with 95% CI.

P-values < 0.05 were considered statistically significant.
